# Prognostic value of PD-L1 expression on tumor-infiltrating immune cells and neutrophil-to-lymphocyte ratio in patients with biliary tract cancer

**DOI:** 10.3389/fimmu.2025.1729542

**Published:** 2026-02-09

**Authors:** Shang Chen, Guizhong Huang, Zehui Yao, Xiaojun Lin, Jianzhong Cao

**Affiliations:** 1 State Key Laboratory of Oncology in South China, Guangdong Provincial Clinical Research Center for Cancer, Sun Yat-sen University Cancer Center, Guangzhou, China; 2 Department of Medical Oncology, Henan Cancer Hospital, Affiliated to Zhengzhou University, Zhengzhou, Henan, China

**Keywords:** BTC, immunotherapy, NLR, PD-L1, TIICs

## Abstract

**Background:**

The expression of Programmed Death-Ligand 1 (PD-L1) on tumor-infiltrating immune cells (TIICs), plays a crucial role in tumor progression and immune evasion, impacting both the natural immune response and immune-targeted therapeutic strategies. The neutrophil-to-lymphocyte ratio (NLR) has also gained attention as a potential predictive biomarker for immunotherapy efficacy, as it may correlate with treatment outcomes.

**Objective:**

To examine the expression of PD-L1 on TIICs and assess the influence of PD-L1 and NLR on immunotherapy outcomes following biliary tract cancers (BTC) recurrence.

**Methods:**

From January 1, 2017, to January 1, 2020, this study enrolled 239 patients from the Department of Pancreaticobiliary Surgery at Sun Yat-sen University Cancer Center. Immunohistochemical analysis of PD-L1 on TIICs was conducted on pathological tissue sections from these patients. Clinical data, including overall survival (OS), disease-free survival (DFS), and pathological findings, were collected during follow-up. Statistical analyses were performed to assess outcomes related to the study objectives. Furthermore, data from The Cancer Genome Atlas (TCGA) were utilized to examine PD-L1 expression profiles and related information.

**Results:**

Tumor stage did not differ significantly (P = 0.173), while metastasis stage approached significance (P = 0.093), with a higher proportion of M0 cases in the PD-L1 low group. Univariate analysis revealed vascular tumor thrombus, tumor differentiation, node stage, and preoperative CA199 levels as factors associated with DFS. Notably, vascular tumor thrombus (HR = 1.791, P = 0.002), moderate tumor differentiation (HR = 0.537, P = 0.002), and elevated preoperative CA199 levels (>35, HR = 1.624, P = 0.009) emerged as significant risk factors. Elevated NLR demonstrated a significant association with reduced DFS (HR = 1.54, p = 0.017 one week prior; HR = 1.70, p = 0.007 one month after) and diminished OS (HR = 2.30, p < 0.001 one week prior; HR = 1.94, p = 0.005 one month after). Exploratory analysis in a limited immunotherapy subgroup (n=35) suggested patients exhibiting high PD-L1 levels on TIICs may be associated with worse OS following immunotherapy after recurrence (HR = 3.03, p = 0.036). High NLR, both one month before recurrence (HR = 2.23, p = 0.015) and one month after recurrence (HR = 2.10, p = 0.027), correlated with decreased OS.

**Conclusion:**

PD-L1 expression on TIICs and dynamic NLR may be indicative of prognosis in BTC and could provide insights into immune status and response to immunotherapy after recurrence. These findings highlight the potential value of integrating local immune contexture with systemic inflammatory markers, but further validation in larger and prospective cohorts is warranted.

## Background

Biliary tract cancers (BTC), a malignant cancer originating from bile duct epithelial cells, is characterized by its aggressive nature and poor prognosis (1). The interaction between tumor cells and the immune system has received significant attention, particularly regarding immune checkpoint molecules such as PD-L1 (2). PD-L1 expression in tumor-associated immune infiltrates is a crucial factor influencing tumor progression and immune evasion, directly affecting the efficacy of immune responses and immune-targeting therapies. PD-L1 is a key component of the immune checkpoint pathway, regulating T-cell activation and modulating immune responses. It interacts with the PDCD1 (PD-1) receptor on T-cells, resulting in the suppression of anti-tumor immune responses (3, 4). In CCA, PD-L1 is expressed by both malignant cells and tumor-infiltrating immune cells. These immune infiltrates primarily include CD8+ cytotoxic T lymphocytes, regulatory T cells (Tregs), and myeloid-derived suppressor cells (MDSCs), all of which play critical roles in shaping the immune landscape of the tumor microenvironment (5, 6). The presence of PD-L1 in these immune infiltrates suggests a complex mechanism through which CCA evades immune surveillance. Increased PD-L1 expression in tumor-associated macrophages (TAMs) and dendritic cells contributes to an immunosuppressive microenvironment, inhibiting the activation and proliferation of intratumoral effector T-cells (7, 8). This process not only hinders the anti-tumor immune response but also correlates with poor clinical outcomes, as numerous studies have demonstrated that high PD-L1 expression is associated with advanced disease stages and reduced overall survival.

Beyond immune evasion, PD-L1 expression holds significant implications for the development of targeted immunotherapies. Theintroduction of immune checkpoint inhibitors has transformed cancer treatment, including for CCA (9, 10). Clinical trials investigating PD-1/PD-L1 blockade in CCA patients have demonstrated varied responses, emphasizing the necessity for a more comprehensive understanding of the tumor microenvironment and PD-L1 dynamics to enhance therapeutic efficacy (11). PD-L1 expression in immune infiltrates may serve as a potential biomarker for predicting a patient’s response to immunotherapy, underscoring the importance of its clinical assessment. Immunotherapy, which leverages the body’s immune system to eradicate cancer cells, has emerged as a promising treatment modality for CCA (12, 13). Among the predictive markers for immunotherapy efficacy, the NLR has garnered attention as a potential biomarker associated with treatment outcomes (14, 15). The NLR, derived from peripheral blood, reflects the balance between neutrophils and lymphocytes, two crucial components of the immune system. Elevated NLR is correlated with poor prognosis in several malignancies, including CCA (16).

The underlying mechanism indicates that an elevated neutrophilic response can generate an immunosuppressive tumor microenvironment, potentially reducing the efficacy of immune-mediated therapies (17). Elevated neutrophil counts result in the release of pro-inflammatory cytokines that promote tumor growth and immune evasion, while lymphocytes, particularly CD8+ T cells, play a crucial role in mounting effective anti-tumor responses. In the context of CCA immunotherapy, research has demonstrated that a higher pre-treatment NLR is associated with reduced overall survival and progression-free survival (18, 19). Patients with low NLR values may demonstrate a more robust baseline immune response, potentially enhancing their response to immune checkpoint inhibitors such as pembrolizumab or nivolumab, which inhibit pathways that suppress T-cell activation. Conversely, a high NLR may indicate an existing inflammatory state that impedes the development of an effective adaptive immune response, hindering immune attacks on tumor cells. Furthermore, monitoring dynamic changes in NLR during immunotherapy can provide insights into treatment efficacy. A decrease in NLR during therapy might suggest a favorable response, indicating effective immune activation against the tumor (20, 21). In contrast, a stable or increasing NLR could prompt clinicians to consider alternative therapeutic options, suggesting that the current immunotherapy regimen may not be benefiting the patient.

## Methods

### Study design

This retrospective study utilized anonymized clinical data collected after obtaining informed consent for treatment from each participant. The research adhered to all relevant legal and ethical guidelines, including the Declaration of Helsinki, and complied with the regulations set by the local Institutional Review Board (IRB) at Sun Yat-sen University Cancer Center (Ethics number B2021-112).

### Study subjects

This study encompassed 239 patients treated at the Department of Pancreatic and Biliary Oncology, Sun Yat-sen University Cancer Center, from January 1, 2017, to January 1, 2020. Pathological tissue sections from these patients underwent immunohistochemical analyses for PD-L1. PD-L1 expression was assessed using two complementary scoring systems: Tumor Proportion Score (TPS) and Combined Positive Score (CPS). TPS represents the percentage of PD-L1-positive tumor cells among all viable tumor cells: TPS = (number of PD-L1 positive tumor cells/total number of tumor cells) × 100%. CPS accounts for PD-L1 expression in both tumor cells and tumor-associated immune cells, providing a more comprehensive assessment of the tumor immune microenvironment: CPS = (number of PD-L1-stained tumor cells + number of PD-L1-stained tumor-associated immune cells)/total number of tumor cells × 100%. PD-L1 positivity on TIICs was defined using the internationally recognized CPS-TPS system. Patients were stratified into high and low TIICs PD-L1 groups according to the median CPS–TPS value of the cohort. A CPS or TPS score of ≥1 was considered positive, following ASCO and CAP guidelines (22, 23). By including immune cell staining, CPS better reflects overall immune activation and the tumor microenvironment and is particularly useful for identifying cholangiocarcinoma patients who may benefit from PD-1/PD-L1 inhibitor therapy. Integrating TPS and CPS thus provides a comprehensive evaluation of PD-L1 as a prognostic and predictive biomarker in cholangiocarcinoma. Tumor tissue sections were deparaffinized, rehydrated, and subjected to antigen retrieval using a high-pH buffer. The staining procedure adhered to the manufacturer’s recommendations. All tissue slides were independently evaluated by two pathologists, and discrepancies were resolved through consensus. Kappa statistical analysis was performed to assess inter-observer agreement, with a Kappa value of ≥0.75 indicating good agreement. Prior to the study, pathologists received standardized training on the scoring system and staining protocol. Regular calibration sessions were held to ensure consistency in scoring throughout the study period. To minimize potential bias, pathologists were blinded to clinical patient data, including treatment regimens and outcomes, during the assessment of PD-L1 expression.

During follow-up, clinical data were collected, including OS, DFS, and pathological findings. The collected data were then subjected to statistical analyses to examine outcomes in relation to the research objectives. Additionally, data from TCGA database were utilized to investigate the expression profiles of PD-L1 and related information. Although no universally accepted cutoff exists for BTC, prior studies have applied thresholds ranging from 2.0 to 5.0 depending on cohort characteristics (24, 25). For survival analyses, NLR was dichotomized into high and low groups using the median value at each evaluated time point. This approach was chosen to minimize bias associated with outcome-driven cutoff selection. ROC analysis was performed separately to assess the discriminative performance of NLR but was not used to define primary cutoff values.

### Inclusion criteria

Patients were retrospectively enrolled based on the following criteria: a confirmed diagnosis of BTC, in accordance with the criteria established by the International Union Against Cancer (UICC), verified by pathological and/or cytological examination; age between 18 and 80 years at the time of diagnosis; and an estimated life expectancy of more than 3 months.

### Exclusion criteria

Exclusion Criteria: Cases were excluded if clinical records were incomplete (e.g., lacking essential diagnostic, treatment, or follow-up data) or if the diagnosis was not unequivocally confirmed via pathological, imaging, or laboratory evaluations. Moreover, patients with severe systemic conditions (such as acute heart failure, end-stage renal or hepatic disease, or other significant comorbidities) were omitted to reduce potential confounding factors. Cases with a history or concurrent diagnosis of other malignancies, an inadequate follow-up period, documented refusal of data use, or unverified abnormal laboratory/imaging findings were also excluded from the study.

### Data collection

Data were collected from a cohort of 239 patients admitted to the Oncology Department between January 1, 2017, and January 1, 2020. All patients underwent radical BTC resection. All patients included in this study underwent surgical resection without preoperative therapy. Postoperative management consisted of adjuvant chemotherapy, and targeted therapy or immunotherapy was administered upon recurrence according to clinical indications. As the primary focus of this study was on immunotherapy outcomes, detailed data on other treatments were not included in the statistical analyses. Due to economic and policy considerations, the immunotherapy drugs used were Chinese-made PD-1 inhibitors, including Toripalimab, Sintilimab, Camrelizumab, and Tislelizumab (dose of 200 mg every 2–3 weeks intravenously). Immunotherapy is mostly used after disease recurrence after surgery for BTC. Clinical information, routine blood tests, and blood biochemistry were obtained. **Immunohistochemistry:** Immunohistochemistry (IHC) was performed using the Epics XL flow cytometer (Coulter, USA). IHC was conducted using the PD-L1 Polyclonal Antibody (Catalog No. PA5-20343, Thermo-Fisher Scientific, RRID: AB_11153819, diluted 1:100). The procedure was carried out using the DAKO Auto-stainer (Model X) following the manufacturer’s instructions. DAKO Chromogen DAB was utilized for color development. The staining procedure was standardized for all samples, with negative controls included in each experiment. The DAKO Auto-stainer ensured consistent, reproducible staining results. Cellular images were captured using a Nikon Eclipse Ni-U microscope (Nikon Instruments, Japan) in both bright-field and fluorescence modes. Images were acquired using a high-resolution camera and analyzed with Nikon NIS-Elements software (version X).

**Evaluation Criteria:** This study analyzed 239 pathological tissue sections. Following immunohistochemical (IHC) analysis, independent pathologists from the Department of Pathology evaluated and scored the slides. The scoring system encompassed three main components: 1) Identification and enumeration of positively stained tumor cells exhibiting partial or complete membrane staining; 2) Identification and quantification of positively stained immune cells, including lymphocytes and macrophages, displaying membrane or cytoplasmic staining; and 3) Enumeration of the total viable tumor cells within each sample.

**Statistical Methods:** Data analysis was conducted using SPSS 22.0 software. For normally distributed continuous data, the mean ± standard deviation was reported. Between-group comparisons were performed using the Student’s t-test. Non-normally distributed data were analyzed using the Kruskal-Wallis test (K-W test), with the median (interquartile range) reported as [M (P25, P75)]. Categorical data were expressed as proportions, and between-group comparisons were conducted using the chi-square test (X² test). Statistically significant differences were defined as P < 0.05.

## Result

### An immunological analysis related to CD274 (PD-L1) was performed using the TCGA database, examining immune cell enrichment, composition, and associations with immune checkpoint markers

**Figure 1:**
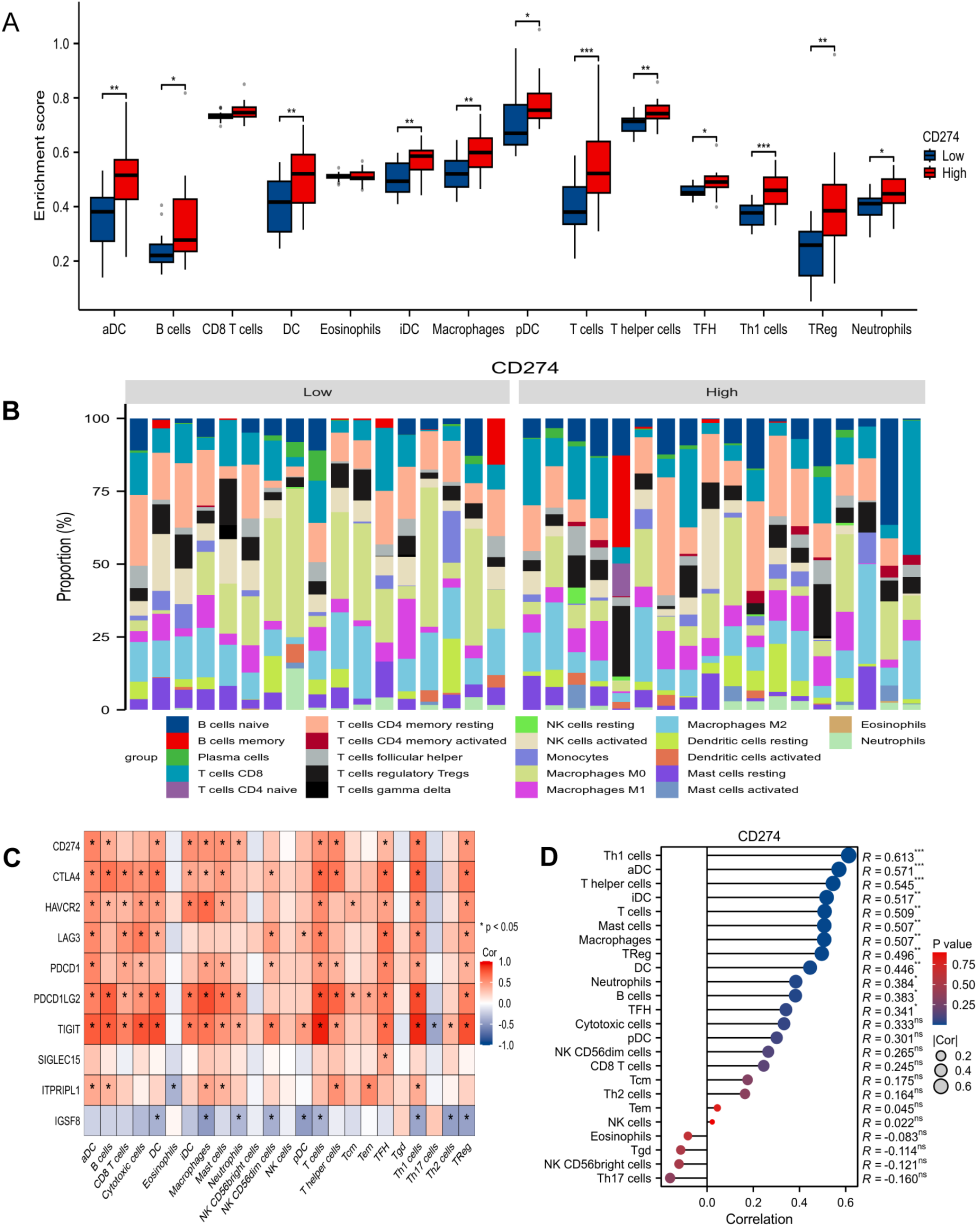
In TCGA database, immunological analysis related to CD274 (PD-L1), focusing on immune cell enrichment, composition, and correlation with immune checkpoint markers. **(A)** Immune Cell Enrichment by CD274 Expression. **(B)** The proportion of different immune cell subtypes in samples with low and high CD274 expression. the proportion of different immune cell subtypes in samples with low and high CD274 expression. **(C)** Heatmap showing correlations between CD274 and other immune checkpoint molecules (e.g., CTLA4, LAG3, TIGIT) and immune cell subtypes. **(D)** Correlation Between CD274 and Immune Cell Types. *p < 0.05, **p < 0.01, ***p < 0.001, ns, not significant.

Panel A illustrates the enrichment scores of various immune cell types in samples with low and high CD274 expression. Significant differences were observed in activated dendritic cells (aDC), B cells, CD8 T cells, dendritic cells (DC), T helper cells, regulatory T cells (TReg), and neutrophils, with high CD274 expression correlating with higher enrichment scores in most immune cell types (*p < 0.05, **p < 0.01, ***p < 0.001). Panel B depicts the proportional composition of immune cell subtypes in samples with low and high CD274 expression, highlighting variations in memory B cells, CD8 T cells, monocytes, macrophages (M0, M1, M2), and resting/activated natural killer (NK) cells. Panel C presents a heatmap displaying the correlation between CD274 and other immune checkpoint markers, including CTLA4, LAG3, PD-1, PVRIG, and TIGIT across various immune cell subtypes. The heatmap reveals significant positive correlations between CD274 and other immune checkpoints, particularly with regulatory T cells, dendritic cells, and macrophages. Panel D shows the correlation coefficients (R values) between CD274 expression and various immune cell types. Notably, CD274 exhibits a strong positive correlation with Th1 cells (R = 0.613), aDC (R = 0.547), and T helper cells (R = 0.531), indicating that higher PD-L1 expression is associated with increased infiltration of specific immune cells in the tumor microenvironment. These findings suggest that CD274 expression is closely linked to immune cell composition and immune checkpoint activity, providing insights into the immune landscape of tumors with high PD-L1 expression (**Figure 1**).

### The expression of PD-L1 on TIICs and its correlation with immune and diagnostic markers

**Figure 2:**
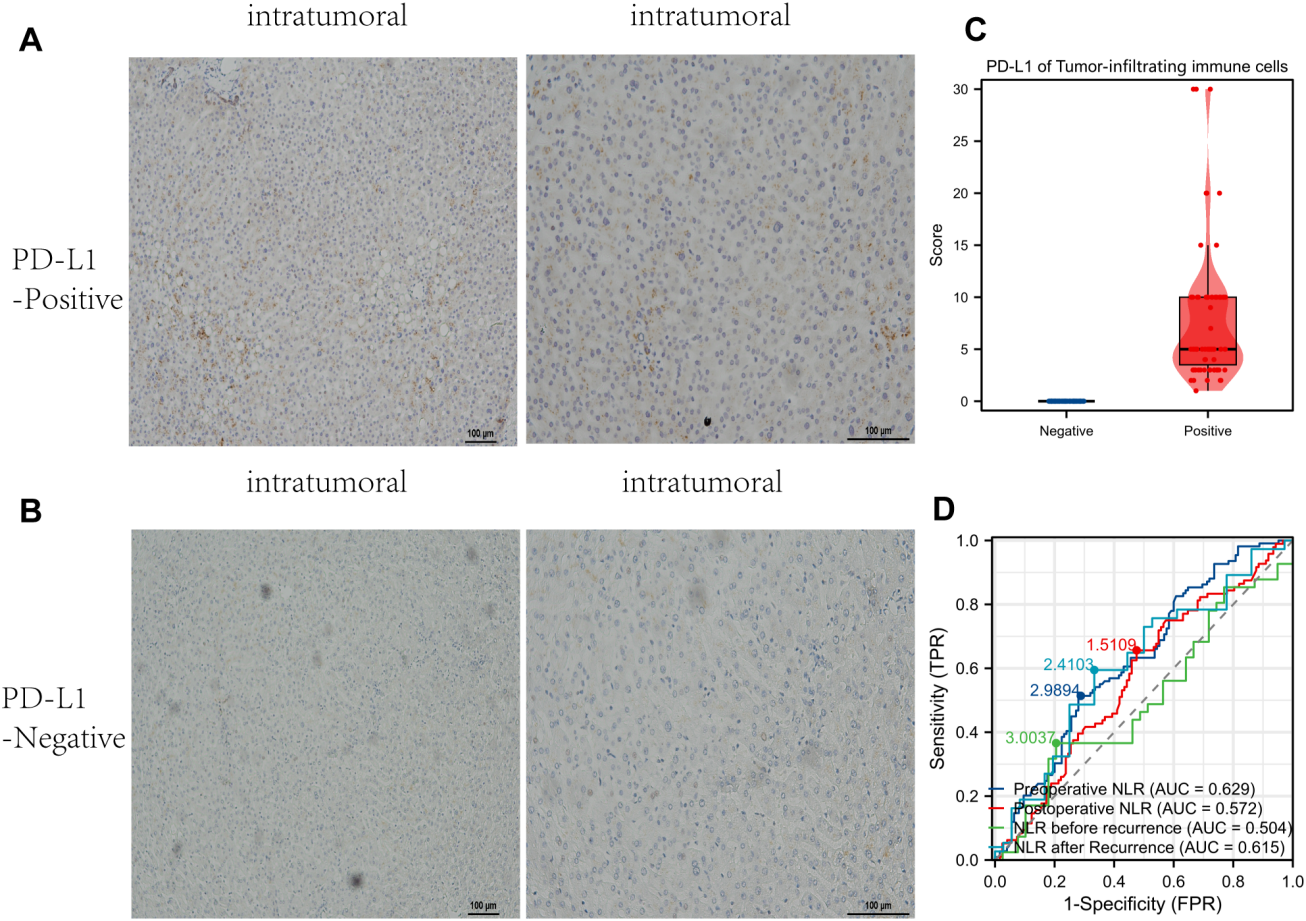
The expression of PD-L1 (CD274) in Intratumoral tissue and its correlation with immune and diagnostic markers. **(A)** Intratumoral Tumor-Infiltrating Immune Cells (TIICs) that are PD-L1 positive. **(B)** Intratumoral Tumor-Infiltrating Immune Cells (TIICs)that are PD-L1 negative. **(C)** A comparison of PD-L1 expression scores on tumor-infiltrating immune cells (TIICs) between PD-L1 positive and negative samples. **(D)** ROC Curves for Neutrophil-to-Lymphocyte Ratio (NLR).

The staining reveals distinct patterns of PD-L1 expression across the examined tissues, with positive samples exhibiting more pronounced brown staining. PD-L1-positive samples demonstrate specific staining in immune or tumor-associated cells, suggesting higher PD-L1 activity in these areas. Conversely, PD-L1-negative samples display lighter staining, suggesting minimal or absent PD-L1 expression in these tissues (**Figures 2A, B**). A comparative analysis of PD-L1 expression scores on TIICs between PD-L1-positive and PD-L1-negative samples is presented. Positive samples demonstrate significantly higher scores compared to negative samples. The elevated scores in PD-L1-positive on TIICs suggest a potentially immunosuppressive or regulatory microenvironment. The wide distribution of scores observed in positive samples indicates variability in PD-L1 expression levels across the dataset (**Figure 2C**). Receiver Operating Characteristic (ROC) curves were employed to assess the diagnostic utility of NLR at various clinical stages: preoperative NLR, postoperative NLR, NLR before recurrence, and NLR after recurrence. Area Under the Curve (AUC) values are provided for each condition. Preoperative NLR (AUC = 0.629) demonstrates moderate predictive power, while postoperative NLR (AUC = 0.572) exhibits slightly lower predictive utility. NLR before recurrence (AUC = 0.504) shows minimal predictive value, whereas NLR after recurrence (AUC = 0.615) regains moderate predictive utility. These findings suggest that NLR serves as a useful biomarker for certain stages but has limited utility in predicting recurrence (**Figure 2D**).

### Patient characteristics and clinical data summary

This section presents a summary of the patient cohort characteristics. The study included 239 patients, comprising 137 (57.3%) males and 102 (42.7%) females. The median age was 62 years, with a range of 54 to 69 years. Intrahepatic cholangiocarcinoma was the most prevalent cancer type (54%), followed by extrahepatic cholangiocarcinoma (19.7%), gallbladder cholangiocarcinoma (16.7%), and hilar cholangiocarcinoma (9.2%). Tumor differentiation analysis revealed that 55.6% of patients had poorly differentiated tumors, 39.3% had moderately differentiated tumors, 4.7% had well-differentiated tumors, and 0.5% had undifferentiated tumors. Vascular tumor thrombus was observed in 29.3% of patients, while 42.7% exhibited perineural invasion. Regarding tumor staging, the majority of patients presented with T1 tumors (44.7%), followed by T2 (31.2%), T3 (19.4%), and T4 (4.6%). Node staging showed 68.4% at N0, 29.5% at N1, and 2.1% at N2. The majority (94.5%) of patients were classified as M0 (non-metastatic), while 5.5% were M1 (metastatic). TNM staging indicated that most patients were at stage II (37%), followed by stage III (27.3%), stage I (28.6%), and stage IV (7.1%). The median preoperative NLR was 2.6312 (range: 1.8383 to 3.5753). The postoperative NLR at one month had a median value of 1.6298 (range: 1.1811 to 2.3077), while the NLR before recurrence was 1.9615 (range: 1.4264 to 3.2035), and the NLR after recurrence increased to 2.171 (range: 1.4266 to 3.4). The median Glasgow liver score was 3 (range: 0–4), the median Glasgow inflammatory grade was 2 (range: 0–2), and the median Glasgow liver fibrosis score was 1 (range: 0–2). Preoperative CA199 levels were ≤35 in 43.1% of patients and >35 in 56.9%. Postoperative CA199 levels were ≤35 in 69.5% and >35 in 30.5% (**Table 1**).

**Table 1:** Patient characteristics.

Characteristics	Overall
Gender, n (%)	
Male	137 (57.3%)
Female	102 (42.7%)
Age(years), n median Range	62 54- 69
Types of cancer, n (%)	
Intrahepatic Cholangiocarcinoma	129 (54%)
Extrahepatic Cholangiocarcinoma	47 (19.7%)
Hilar Cholangiocarcinoma	22 (9.2%)
Gallbladder Cholangiocarcinoma	40 (16.7%)
Degree of differentiation, n (%)	
Poorly Differentiated	119 (55.6%)
Moderately Differentiated	84 (39.3%)
Well Differentiated	10 (4.7%)
Undifferentiated	1 (0.5%)
Vascular tumor thrombus, n (%)	
no	169 (70.7%)
yes	70 (29.3%)
Perineural invasion, n (%)	
no	137 (57.3%)
yes	102 (42.7%)
Tumor stage, n (%)	
T1	106 (44.7%)
T2	74 (31.2%)
T3	46 (19.4%)
T4	11 (4.6%)
Node stage, n (%)	
N0	162 (68.4%)
N1	70 (29.5%)
N2	5 (2.1%)
Metastasis stage, n (%)	
M0	225 (94.5%)
M1	13 (5.5%)
TNM Stage, n (%)	
II	88 (37%)
III	65 (27.3%)
I	68 (28.6%)
IV	17 (7.1%)
1week Preoperative NLR, median	2.6312 (1.8383, 3.5753)
1month Postoperative NLR, median	1.6298 (1.1811, 2.3077)
Before recurrence NLR, median (IQR)	1.9615 (1.4264, 3.2035)
After recurrence NLR, median (IQR)	2.171 (1.4266, 3.4)
Glasgow Liver Score, median (IQR)	3 (0, 4)
Glasgow Inflammatory Grad, median (IQR)	2 (0, 2)
Glasgow liver fibrosis Score, median (IQR)	1 (0, 2)
Preoperative CA99, n (%)	
<=35	103 (43.1%)
>35	136 (56.9%)
Postoperative CA199, n (%)	
<=35	166 (69.5%)
>35	73 (30.5%)

### Clinicopathological characteristics stratified by PD-L1 expression in tumor-infiltrating immune cells

The clinicopathological characteristics of patients were stratified according to PD-L1 expression in TIICs. A comparison between the low and high PD-L1 expression groups revealed no significant differences in gender distribution (P = 0.523), age (P = 0.214), or cancer type (P = 0.366). In both groups, intrahepatic cholangiocarcinoma was predominant; however, a higher proportion of patients with low PD-L1 expression had other cancer types. No significant differences were observed in tumor differentiation between the groups (P = 0.343), with poorly differentiated cancers being the most prevalent in both. Similarly, vascular tumor thrombus (P = 0.514) and node stage (P = 0.769) did not differ significantly between the groups. A significant difference was observed in perineural invasion (P = 0.042), with a higher incidence in the high PD-L1 expression group. While tumor stage did not significantly differ (P = 0.173), metastasis stage approached significance (P = 0.093), with more M0 cases in the low PD-L1 group. Other clinical factors, including preoperative and postoperative NLR (P = 0.916 and P = 0.068, respectively), Glasgow scores (P = 0.141 for liver score, P = 0.288 for inflammatory grade, P = 0.118 for liver fibrosis), and CA199 levels (P = 0.653 for preoperative, P = 0.515 for postoperative), did not show significant differences between the two PD-L1 groups. These findings suggest that PD-L1 expression in TIICs is not strongly associated with most clinicopathological features, although differences in perineural invasion and metastasis stage were observed between the groups (**Table 2**).

**Table 2:** Clinicopathological characteristics stratified by PD-L1 of tumor-infiltrating immune cells (TIICs) expression.

Characteristics	PD-L1/low	PD-L/high	P value
n	161	78	
Gender, n (%)			0.523
Male	90 (37.7%)	47 (19.7%)	
Female	71 (29.7%)	31 (13%)	
Age (years), median (IQR)	62 (54, 69)	64.5 (56, 68.75)	0.214
Types of cancer, n (%)			0.366
Intrahepatic Cholangiocarcinoma	88 (36.8%)	41 (17.2%)	
Extrahepatic Cholangiocarcinoma	35 (14.6%)	12 (5%)	
Hilar Cholangiocarcinoma	13 (5.4%)	9 (3.8%)	
Gallbladder Cholangiocarcinoma	25 (10.5%)	15 (6.3%)	
Degree of differentiation, n (%)			0.343
Poorly Differentiated	76 (35.5%)	43 (20.1%)	
Moderately Differentiated	60 (28%)	24 (11.2%)	
Well Differentiated	7 (3.3%)	3 (1.4%)	
Undifferentiated	0 (0%)	1 (0.5%)	
Vascular tumor thrombus, n (%)			0.514
no	116 (48.5%)	53 (22.2%)	
yes	45 (18.8%)	25 (10.5%)	
Perineural invasion, n (%)			0.042
no	85 (35.6%)	52 (21.8%)	
yes	76 (31.8%)	26 (10.9%)	
Tumor stage, n (%)			0.173
T1	65 (27.4%)	41 (17.3%)	
T2	51 (21.5%)	23 (9.7%)	
T3	33 (13.9%)	13 (5.5%)	
T4	10 (4.2%)	1 (0.4%)	
Node stage, n (%)			0.769
N0	107 (45.1%)	55 (23.2%)	
N1	48 (20.3%)	22 (9.3%)	
N2	4 (1.7%)	1 (0.4%)	
Metastasis stage, n (%)			0.093
M0	148 (62.2%)	77 (32.4%)	
M1	12 (5%)	1 (0.4%)	
TNM Stage, n (%)			0.104
I	53 (22.3%)	35 (14.7%)	
II	47 (19.7%)	18 (7.6%)	
III	45 (18.9%)	23 (9.7%)	
IV	15 (6.3%)	2 (0.8%)	
Preoperative NLR, median (IQR)	2.6235 (1.786, 3.6783)	2.6363 (1.8853, 3.5172)	0.916
Postoperative NLR, n (%), median (IQR)	1.7377 (1.223, 2.4767)	1.4502 (1.1275, 2.0169)	0.068
Before tumor recurrence NLR, median (IQR)	1.956 (1.4957, 2.3711)	2.809 (1.4063, 4.7821)	0.367
After tumor recurrence NLR, median (IQR)	2.1466 (1.5928, 3.7273)	2.5033 (1.2236, 3.1938)	0.870
Glasgow liver score, median (IQR)	3 (0, 4)	3 (0, 4)	0.141
Glasgow inflammatory grad, median (IQR)	2 (0, 2)	2 (0, 2)	0.288
Glasgow liver fibrosis score, median (IQR)	1 (0, 2)	1 (0, 2)	0.118
Preoperative ca199, n (%)			0.653
≤35	71 (29.7%)	32 (13.4%)	
>35	90 (37.7%)	46 (19.2%)	
Postoperative ca199, n (%)			0.515
≤35	114 (47.7%)	52 (21.8%)	
>35	47 (19.7%)	26 (10.9%)	

### Summary of univariate and multivariate analysis for OS

The univariate and multivariate analyses identified several factors associated with OS in patients with BTC. In the univariate analysis, vascular tumor thrombus, perineural invasion, tumor stage, node stage, metastasis, and preoperative CA199 levels were significantly associated with OS. However, after adjusting for potential confounders in the multivariate analysis, only metastasis (M1) and preoperative CA199 levels emerged as strong independent predictors of OS. Specifically, metastasis (M1) was associated with an increased risk of mortality in both the univariate (HR = 2.300, P = 0.017) and multivariate analyses (HR = 3.029, P = 0.009), while elevated preoperative CA199 levels (>35) were also associated with poorer survival (univariate HR = 2.730, P < 0.001; multivariate HR = 2.216, P < 0.001). Conversely, PD-L1 expression in TIICs did not demonstrate a significant impact on survival (HR = 1.094, P = 0.656), indicating that it may not be a crucial factor for prognosis in these patients. Other factors, including vascular tumor thrombus, perineural invasion, and tumor stage, exhibited varying degrees of significance but did not remain strong predictors after multivariate adjustment. These findings underscore the importance of metastasis and CA199 levels as key prognostic factors in BTC (**Table 3**).

**Table 3:** Summary of univariate and multivariate analysis for overall survival (OS).

Characteristics	Total (N)	Univariate analysis		Multivariate analysis	
		Hazard ratio (95% CI)	P value	Hazard ratio (95% CI)	P value
Gender	227				
Male	129	Reference			
Female	98	0.907 (0.621 - 1.325)	0.613		
Age(years)	227	1.014 (0.994 - 1.034)	0.161		
Degree of differentiation	203				
Poorly Differentiated	113	Reference			
Moderately Differentiated	81	0.819 (0.543 - 1.233)	0.338		
Well Differentiated	9	0.314 (0.077 - 1.287)	0.108		
Vascular tumor thrombus	227				
no	160	Reference		Reference	
yes	67	1.857 (1.267 - 2.722)	0.002	1.344 (0.878 - 2.056)	0.173
Perineural invasion	227				
no	133	Reference		Reference	
yes	94	2.090 (1.435 - 3.044)	< 0.001	1.473 (0.965 - 2.249)	0.073
Tumor stage	225				
T1	99	Reference		Reference	
T2	71	1.967 (1.267 - 3.055)	0.003	1.518 (0.956 - 2.413)	0.077
T3	45	1.956 (1.172 - 3.264)	0.010	1.443 (0.843 - 2.468)	0.181
T4	10	1.831 (0.720 - 4.655)	0.204	0.853 (0.286 - 2.547)	0.776
Node stage	225				
N0	154	Reference		Reference	
N1	66	2.657 (1.809 - 3.900)	< 0.001	1.576 (0.999 - 2.487)	0.050
N2	5	1.063 (0.260 - 4.352)	0.932	0.650 (0.145 - 2.917)	0.573
Metastasis stage	226				
M0	213	Reference		Reference	
M1	13	2.300 (1.161 - 4.556)	0.017	3.029 (1.313 - 6.989)	0.009
Preoperative NLR	225	1.008 (0.991 - 1.026)	0.332		
Postoperative NLR	211	1.032 (0.952 - 1.118)	0.447		
PD-L1 of TIICs	227				
low	153	Reference			
high	74	1.094 (0.737 - 1.622)	0.656		
Glasgow liver score	147	0.945 (0.851 - 1.050)	0.295		
Glasgow inflammatory grad	147	0.883 (0.721 - 1.081)	0.229		
Preoperative ca199	227				
≤35	99	Reference		Reference	
>35	128	2.730 (1.797 - 4.147)	< 0.001	2.216 (1.391 - 3.530)	< 0.001
Postoperative ca199	227				
≤35	157	Reference		Reference	
>35	70	1.865 (1.273 - 2.732)	0.001	1.256 (0.830 - 1.903)	0.281

### Summary of univariate and multivariate analysis for DFS

The univariate and multivariate analyses for DFS revealed several factors significantly influencing outcomes. In the univariate analysis, vascular tumor thrombus, degree of differentiation, node stage, and preoperative CA199 levels were significantly associated with DFS. Specifically, vascular tumor thrombus (HR = 1.791, P = 0.002), moderately differentiated tumors (HR = 0.537, P = 0.002), and elevated preoperative CA199 levels (>35, HR = 1.624, P = 0.009) were identified as significant risk factors for DFS. However, the multivariate analysis identified only degree of differentiation and preoperative CA199 levels as significant predictors of DFS. Moderately differentiated tumors (HR = 0.514, P = 0.002) and well-differentiated tumors (HR = 0.234, P = 0.016) were associated with improved DFS outcomes, while elevated preoperative CA199 levels (>35, HR = 1.784, P = 0.004) correlated with poorer DFS outcomes. Other factors, including vascular tumor thrombus, node stage, metastasis, and PD-L1 expression in TIICs, did not maintain significance after adjusting for confounders. These results indicate that tumor differentiation and preoperative CA199 levels are critical factors influencing disease-free survival in patients with BTC (**Table 4**).

**Table 4:** Summary of univariate and multivariate analysis for disease-free survival (DFS).

Characteristics	Total (N)	Univariate analysis		Multivariate analysis	
		Hazard ratio (95% CI)	P value	Hazard ratio (95% CI)	P value
Gender	239				
Male	137	Reference		Reference	
Female	102	0.706 (0.488 - 1.022)	0.065	0.857 (0.565 - 1.300)	0.467
Age(years)	239	0.990 (0.973 - 1.008)	0.264		
Degree of differentiation	213				
Poorly Differentiated	119	Reference		Reference	
Moderately Differentiated	84	0.537 (0.359 - 0.803)	0.002	0.514 (0.337 - 0.784)	0.002
Well Differentiated	10	0.296 (0.093 - 0.940)	0.039	0.234 (0.072 - 0.761)	0.016
Vascular tumor thrombus	239				
no	169	Reference		Reference	
yes	70	1.791 (1.231 - 2.605)	0.002	1.306 (0.806 - 2.116)	0.278
Perineural invasion	239				
no	137	Reference			
yes	102	1.196 (0.834 - 1.715)	0.331		
Tumor stage	237				
T1	106	Reference		Reference	
T2	74	1.810 (1.212 - 2.705)	0.004	1.516 (0.975 - 2.355)	0.064
T3	46	1.435 (0.870 - 2.368)	0.157	0.943 (0.537 - 1.655)	0.837
T4	11	1.869 (0.745 - 4.687)	0.182	1.505 (0.518 - 4.369)	0.453
Node stage	237				
N0	162	Reference		Reference	
N1	70	1.919 (1.299 - 2.834)	0.001	1.474 (0.893 - 2.433)	0.129
N2	5	1.202 (0.379 - 3.808)	0.754	0.578 (0.156 - 2.134)	0.410
Metastasis stage	238				
M0	225	Reference			
M1	13	1.790 (0.660 - 4.856)	0.253		
Preoperative NLR	234	1.005 (0.988 - 1.023)	0.540		
Postoperative NLR	219	1.032 (0.962 - 1.107)	0.378		
PD-L1 of TIICs	239				
low	161	Reference			
high	78	0.978 (0.673 - 1.422)	0.907		
Glasgow liver score	153	0.982 (0.895 - 1.076)	0.692		
Glasgow inflammatory grad	153	0.945 (0.792 - 1.128)	0.534		
Preoperative ca199	236				
≤35	103	Reference		Reference	
>35	133	1.624 (1.131 - 2.332)	0.009	1.784 (1.208 - 2.634)	0.004
Postoperative ca199	236				
≤35	165	Reference			
>35	71	1.343 (0.917 - 1.965)	0.129		

### Kaplan-Meier survival analyses evaluating the influence of diverse clinical and biological factors on DFS and OS over time (in months)

**Figure 3:**
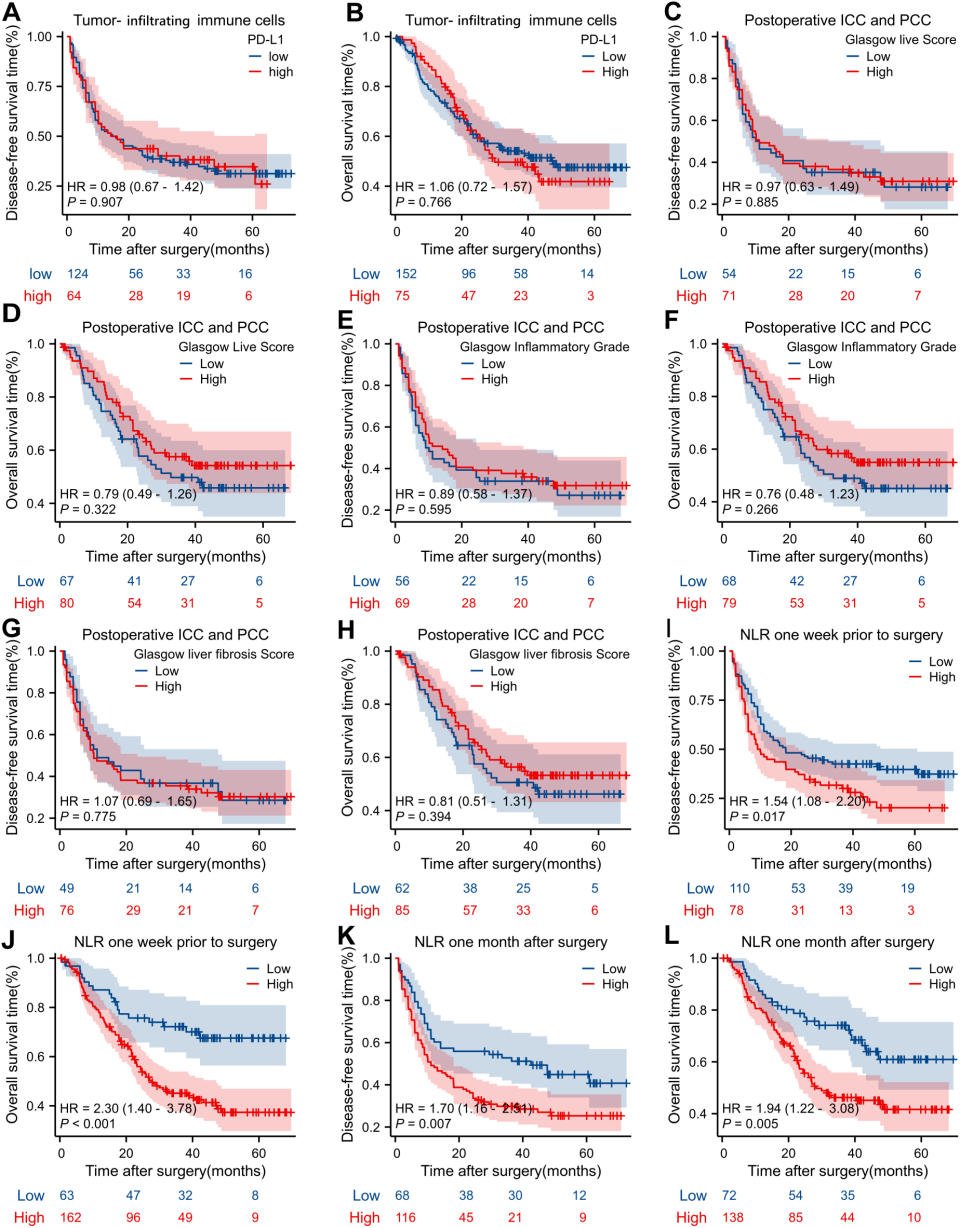
Kaplan-Meier survival analyses assessing the impact of various clinical and biological factors on disease-free survival (DFS) and overall survival (OS) over time. **(A)** The DFS of biliary tract cancers (BTC) tumor-infiltrating immune cells (TIICs) between LD-L1/High and PD-L1/Low. **(B)** The OS of BTC tumor-infiltrating immune cells (TIICs) between LD-L1/High and PD-L1/Low. **(C)** The DFS of Glasgow live score between High and Low in ICC (Intrahepatic cholangiocarcinoma), PCC (Peri-Hilar cholangiocarcinoma). **(D)** The OS of Glasgow live score between High and Low in ICC (Intrahepatic cholangiocarcinoma), PCC (Peri-Hilar cholangiocarcinoma). **(E)** The DFS of Glasgow inflammatory grad between High and Low in ICC (Intrahepatic cholangiocarcinoma), PCC (Peri-Hilar cholangiocarcinoma). **(F)** The OS of Glasgow inflammatory grad between High and Low in ICC (Intrahepatic cholangiocarcinoma), PCC (Peri-Hilar cholangiocarcinoma). **(G)** The DFS of Glasgow fibrosis score between High and Low in ICC (Intrahepatic cholangiocarcinoma), PCC (Peri-Hilar cholangiocarcinoma). **(H)** The OS of Glasgow fibrosis score between High and Low in ICC (Intrahepatic cholangiocarcinoma), PCC (Peri-Hilar cholangiocarcinoma). **(I)** The DFS of Neutrophil-to-Lymphocyte Ratio (NLR) between High and Low in one week prior to surgery. **(J)** The OS of Neutrophil-to-Lymphocyte Ratio (NLR) between High and Low in one week prior to surgery. **(K)** The DFS of Neutrophil-to-Lymphocyte Ratio (NLR) between High and Low in one month after to surgery. **(L)** The OS of Neutrophil-to-Lymphocyte Ratio (NLR) between High and Low in one month after to surgery.

Kaplan-Meier survival curves are presented to analyze the impact of various clinical and biological factors on DFS and OS in patients with BTC. Panels A and B compare DFS and OS between patients with high and low PD-L1 expression on TIICs, revealing no significant differences (HR = 0.98, p = 0.907 for DFS; HR = 1.06, p = 0.766 for OS). Panels C to H assess DFS and OS based on the Glasgow Liver Score, Glasgow Inflammatory Grade, and Glasgow Fibrosis Score ICC and peri-hilar (PCC) cholangiocarcinoma patients. These analyses demonstrate no significant survival differences between the high and low score groups (all p > 0.05). Panels I to L evaluate the NLR one week prior to, and one month after surgery. Elevated NLR is significantly associated with shorter DFS (HR = 1.54, p = 0.017 for one week prior; HR = 1.70, p = 0.007 for one month after) and reduced OS (HR = 2.30, p < 0.001 for one week prior; HR = 1.94, p = 0.005 for one month after). These findings indicate that NLR may serve as a significant prognostic marker in BTC, while PD-L1 expression and Glasgow scores exhibit limited prognostic value (**Figure 3**).

### Kaplan-Meier curve analysis of overall survival time for patients with CCA under different treatment conditions

**Figure 4:**
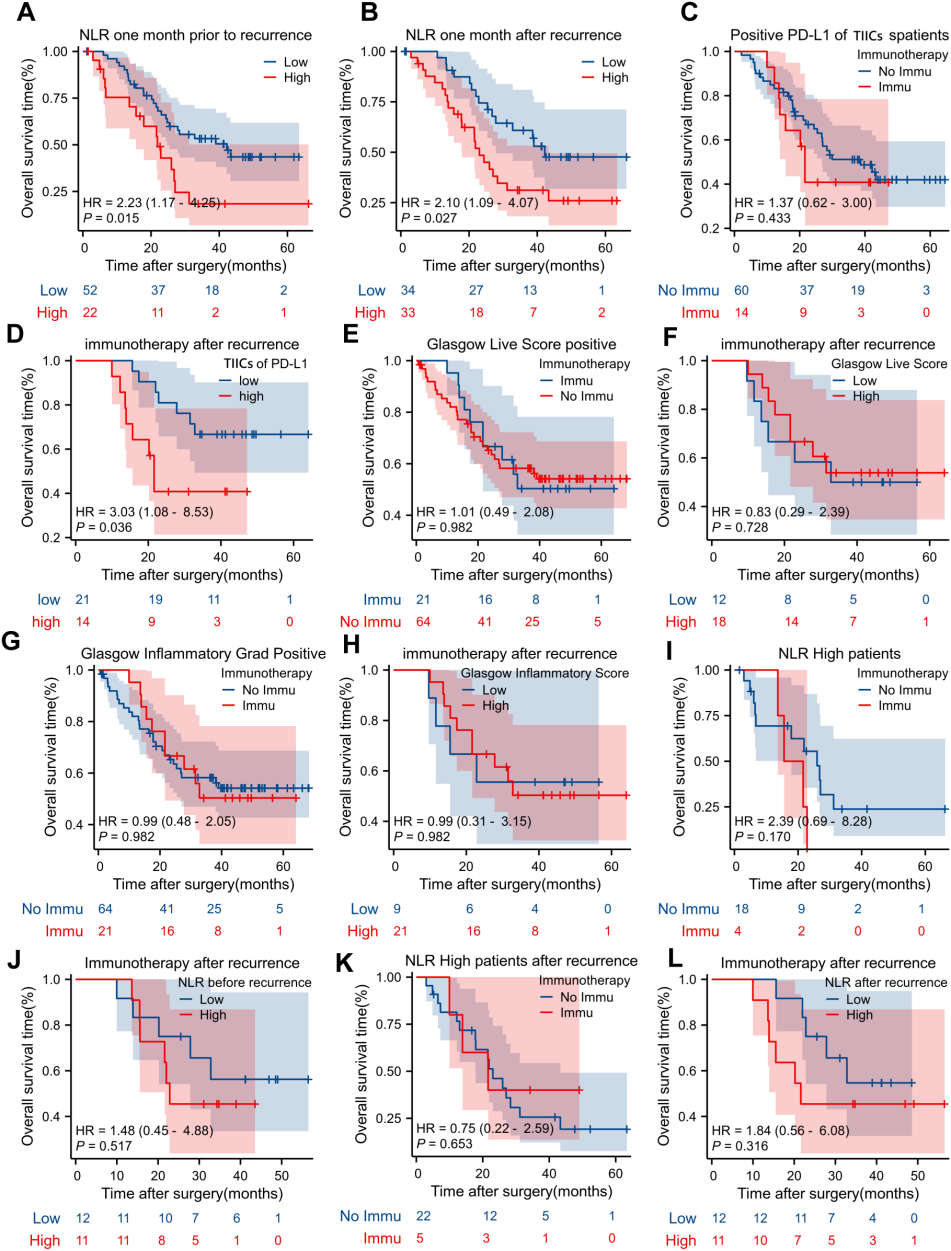
Kaplan-Meier (KM) curve analysis of overall survival time for patients with biliary tract cancers (BTC) under different treatment conditions. **(A)** Compares the OS of patients with low and high expression of Nerve Lymphatic Reflex (NLR) one month prior to recurrence. **(B)** Compares the OS of patients with low and high expression of NLR one month after recurrence. **(C)** Compares the OS of patients with positive PD-L1 in tumor-infiltrating immune cells (TIICs) who received immunotherapy or not. **(D)** Compares the OS of patients who received immunotherapy after recurrence with low and high levels of tumor-infiltrating immune cells (TIICs) of PD-L1. **(E)** Compares OS of patients with positive and negative Glasgow Live Score who received immunotherapy after recurrence. **(F)** Compares the OS of patients with positive and negative Glasgow Live Score who did not receive immunotherapy after recurrence. **(G)** Compares the OS of patients who received immunotherapy after recurrence with positive and negative Glasgow Inflammatory Grade Score. **(H)** Compares the OS of patients who received immunotherapy after recurrence with positive and negative Glasgow Inflammatory Score. **(I)** Compares the OS of NLR high patients who received and did not receive immunotherapy after recurrence. **(J)** Compares OS of patients who received immunotherapy after recurrence, with low NLR and high NLR before recurrence. **(K)** Compares OS of NLR high patients who received immunotherapy after recurrence with not received immunotherapy after recurrence. **(L)** Compares OS of patients who received immunotherapy after recurrence, high and low NLR after recurrence.

Kaplan-Meier survival analyses of OS in CCA patients under various clinical and treatment conditions are presented, emphasizing the NLR, PD-L1 expression in TIICs, and the Glasgow scoring systems in relation to immunotherapy. Panels A and B demonstrate that elevated NLR, both one month prior to recurrence (HR = 2.23, p = 0.015) and one month after recurrence (HR = 2.10, p = 0.027), correlates with inferior OS outcomes. Panel C compares OS in patients with positive PD-L1 expression in TIICs who underwent immunotherapy versus those who did not, indicating no significant difference (HR = 1.37, p = 0.433). Exploratory analysis in the post-recurrence immunotherapy subgroup (n=35) suggested that high PD-L1 expression on TIICs may be associated with inferior overall survival (HR = 3.03, 95% CI 1.08–8.53, P = 0.036). However, the small sample size and wide confidence interval preclude definitive conclusions. Panels E to H evaluate the impact of the Glasgow Liver Score and Glasgow Inflammatory Score on OS, with or without immunotherapy after recurrence, revealing no significant survival differences across these groups (all p > 0.05). Panel I compares OS in high NLR patients who received or did not receive immunotherapy after recurrence, showing no significant difference (HR = 2.39, p = 0.170). Panels J to L further assess the effects of immunotherapy on OS in patients with high or low NLR before and after recurrence, revealing no statistically significant differences (all p > 0.05). These findings suggest that elevated NLR is associated with poorer survival, while the impact of immunotherapy on OS remains inconclusive, necessitating further investigation into its role in CCA management (**Figure 4**).

## Discussion

The examination of PD-L1 expression in TIICs and the influence of the NLR on immunotherapy outcomes following BTC recurrence presents a complex landscape with potential implications for therapeutic strategies. Tumors exhibiting high PD-L1 expression generate an intricate immune microenvironment, characterized by a delicate balance between immune activation and suppression. This duality holds particular relevance in malignancies such as BTC, where the tumor’s capacity to evade immune surveillance plays a crucial role in its progression (26, 27). PD-L1 is frequently upregulated in tumor cells or immune cells within the tumor microenvironment, serving as a mechanism to evade T cell-mediated immune responses, although direct comparison with matched normal tissue was not performed in the full cohort. As a result, PD-L1 inhibition has emerged as a promising therapeutic approach, although its efficacy in BTC remains under investigation, particularly in cases of recurrence (28, 29).

In our cohort, high PD-L1 expression on TIICs was associated with inferior survival following immunotherapy, which may reflect a distinct immune regulatory mechanism compared with tumor cell–intrinsic PD-L1, but the immunotherapy subgroup analysis is limited by the small number of patients (n=35) and should be considered hypothesis-generating. The observed trend toward worse survival in the high PD-L1 group requires validation in larger, prospective cohorts. Recent data in biliary tract cancers suggest that PD-L1 expression localized to immune or stromal compartments is associated with an immunosuppressive microenvironment rather than adaptive IFN-γ-driven activation, and may predict adverse outcomes (2, 30). Mechanistically, PD-L1 expression on myeloid populations, including tumor-associated macrophages and myeloid-derived suppressor cells, can inhibit T-cell proliferation, cytokine production, and cytotoxicity, thereby promoting a state of terminal exhaustion that is refractory to immune checkpoint blockade (31). Therefore, PD-L1 on TIICs may serve as a surrogate of chronic inflammation–driven myeloid immunosuppression and impaired T-cell competence, potentially explaining reduced therapeutic benefit in patients with high expression levels.

In addition to PD-L1 expression, the NLR has emerged as a significant prognostic biomarker reflecting systemic inflammation and immune dysfunction in BTC. An elevated NLR, indicative of a pro-inflammatory state, has been associated with poorer survival outcomes and may adversely affect immunotherapy efficacy (32). The relationship between NLR and immunotherapy is particularly noteworthy, as inflammation has been demonstrated to influence the tumor microenvironment by recruiting pro-tumorigenic immune cells, potentially reducing the effectiveness of immune checkpoint inhibitors. The potential of NLR as a biomarker to predict immunotherapy responses emphasizes the necessity for further research to elucidate its role in both local and systemic immune responses in BTC (33, 34).

The investigation NLR is still in its nascent stages. Although significant correlations have been observed between these factors and patient outcomes, their precise role in predicting immunotherapy efficacy remains ambiguous. The variability in individual patient responses to immunotherapy, coupled with the heterogeneity of the tumor microenvironment, complicates the utilization of these markers as reliable predictors. Furthermore, the interaction between NLR and PD-L1 expression remains insufficiently explored (35, 36). It is conceivable that an elevated NLR could modulate the immune response in a manner that attenuates the effectiveness of PD-L1 inhibitors; however, additional research is necessary to substantiate this hypothesis.

A more comprehensive understanding of the relationship between NLR, tumor immunogenicity, and immune cell recruitment and activation could potentially enhance NLR’s reliability as a predictive biomarker for BTC treatment. For example, combining NLR with other immunological parameters, such as tumor mutational burden (TMB) and microsatellite instability (MSI), might provide a more precise assessment of a patient’s potential response to immunotherapy (37, 38). Furthermore, incorporating additional inflammatory markers, such as C-reactive protein (CRP), with NLR could offer a more holistic view of the inflammatory status and immune response. Further investigation is necessary to elucidate the mechanisms by which systemic inflammation, as measured by NLR, influences the tumor immune microenvironment and impacts treatment outcomes (39, 40). Clinical trials evaluating the effectiveness of combining immunotherapy with strategies to mitigate systemic inflammation may be particularly advantageous for patients with elevated NLR, who might exhibit suboptimal responses to conventional immune checkpoint inhibitors. Moreover, approaches aimed at modulating the tumor immune microenvironment, such as combination therapies incorporating PD-L1 inhibitors and other immunomodulatory agents, could potentially unveil novel pathways for enhancing outcomes in BTC (41, 42).

In conclusion, the NLR plays a crucial role in determining the response to immunotherapy in BTC. Elevated PD-L1 levels may suggest immune evasion and a poor response to immunotherapy,

while a high NLR suggests a systemic inflammatory environment that could impair immune response effectiveness. Understanding the intricate relationship between these factors will inform therapeutic strategies, particularly for recurrent BTC, where immunotherapy has demonstrated both benefits and limitations. As research progresses, incorporating these markers into clinical practice will be essential for patient stratification and optimization of personalized treatment plans. This approach has the potential to enhance survival outcomes and quality of life for patients with this challenging malignancy. The development of combination therapies targeting both immune checkpoints and the inflammatory microenvironment presents a promising avenue for improving the efficacy of immunotherapeutic interventions in BTC. In BTC, patients with elevated NLR and high PD-L1 expression may represent a distinct high-risk subgroup that could benefit from intensified or tailored treatment strategies. Moreover, incorporating NLR and PD-L1 into clinical predictive models could enhance patient stratification and facilitate personalized therapeutic approaches. We advocate for the design of prospective clinical trials specifically in BTC to validate the predictive utility of these biomarkers in guiding treatment decisions, ultimately optimizing treatment regimens and potentially improving overall survival. But This study did not collect data on immune-related adverse events (irAEs). Emerging evidence shows irAEs correlate with improved outcomes in ICI therapy, likely reflecting robust T-cell activation (43). Future prospective studies should include irAE assessment to validate PD-L1/NLR predictive value in this context. We did not adjust for active infections or steroid use, which can significantly confound NLR and obscure its prognostic value (44). This limitation should be addressed in future prospective studies. While PD-L1 and NLR have been studied in BTC, previous reports focused mainly on tumor-cell PD-L1 or advanced disease. In contrast, our study specifically examines PD-L1 on tumor-infiltrating immune cells in resected specimens and integrates this with NLR as a combined immune-inflammatory prognostic signature. Our exploratory immunotherapy subgroup analysis further suggests potential resistance mechanisms associated with high immune-cell PD-L1.

### Limitations

The immune microenvironment is dynamic, and PD-L1 expression at the time of primary surgery may not fully represent the immune status at recurrence when immunotherapy is administered. This temporal discrepancy represents an important limitation of our study and underscores the need for prospective studies incorporating longitudinal immune profiling with repeat biopsies at recurrence. Although all patients received surgery and postoperative treatments were recorded, detailed information on chemotherapy or targeted therapies was not included in the analyses. Therefore, the potential influence of these treatments on survival outcomes could not be fully assessed. Future studies with comprehensive treatment data are warranted to further clarify the impact of immunotherapy in this patient population. The absence of PD-L1 assessment in adjacent non-tumoral tissue, due to limited archival material, limits definitive confirmation of tumor-specificity for elevated TIICs PD-L1 expression.

## Data Availability

The raw data supporting the conclusions of this article will be made available by the authors, without undue reservation.
